# Alternative cleavage and polyadenylation of genes associated with protein turnover and mitochondrial function are deregulated in Parkinson’s, Alzheimer’s and ALS disease

**DOI:** 10.1186/s12920-019-0509-4

**Published:** 2019-05-09

**Authors:** Radhika Patel, Cillian Brophy, Mark Hickling, Jonathan Neve, André Furger

**Affiliations:** Department of Biochemistry, University of Oxford, Oxford, OX1 3QU UK

**Keywords:** Alternative polyadenylation, Neurodegenerative disease

## Abstract

**Background:**

Transcriptome wide changes have been assessed extensively during the progression of neurodegenerative diseases. Alternative polyadenylation (APA) occurs in over 70% of human protein coding genes and it has recently been recognised as a critical regulator of gene expression during disease. However, the effect of APA in the context of neurodegenerative diseases, to date, has not been widely investigated. Dynamic Analysis of Alternative Polyadenylation from RNA-seq (DaPars) is a method by Xia and colleagues [Nat Commun. 5:5274, 2014] to investigate APA using standard RNA-seq data. Here, we employed this method to interrogate APA using publicly available RNA-seq data from Alzheimer’s disease (AD), Parkinson’s disease (PD) and Amyotrophic Lateral Sclerosis (ALS) patients and matched healthy individuals.

**Results:**

For all three diseases, we found that APA profile changes were limited to a relative small number of genes suggesting that APA is not globally deregulated in neurodegenerative disease. However, for each disease phenotype we identified a subgroup of genes that showed disease-specific deregulation of APA. Whilst the affected genes differ between the RNA-seq datasets, in each cohort we identified an overrepresentation of genes that are associated with protein turnover pathways and mitochondrial function.

**Conclusions:**

Our findings, while drawn from a relatively small sample size, suggest that deregulation of APA may play a significant role in neurodegeneration by altering the expression of genes including *UBR1* and *OGDHL* in AD, *LONP1* in PD and *UCHL1* in ALS. This report thus provides important novel insights into how APA can shape neurodegenerative disease characteristic transcriptomes.

**Electronic supplementary material:**

The online version of this article (10.1186/s12920-019-0509-4) contains supplementary material, which is available to authorized users.

## Background

Neurodegeneration is defined as the progressive loss of neurons in the central nervous system (CNS) and the brain. While neurodegenerative diseases can be inherited, these familial cases account for fewer than 10% for the three most common diseases: Alzheimer’s Disease (AD), Parkinson’s Disease (PD) and Amyotrophic Lateral Sclerosis (ALS). Interestingly, whilst the cause of disease in sporadic patients is largely unknown, both the sporadic (non-inherited) and familial cases of a particular neurodegenerative disease often exhibit the same symptoms.

Despite AD, PD and ALS exhibiting very different symptoms and affecting different parts of the CNS, many similarities have been identified at the cellular level, that contribute to neuronal loss. This includes compromised mitochondrial function and protein aggregation to form plaques or inclusion bodies that impair neuronal function [1]. Protein aggregates, often consisting of misfolded proteins, are usually degraded via the ubiquitin proteasome pathway (UPP) or through the autophagy pathway. Deregulation of these pathways is associated with neurodegeneration. However, it is unclear if deregulation of protein degradation pathways is a cause or consequence of neurodegeneration [2].

Transcripts encoding all metazoan protein coding genes, apart from replication dependant histone genes, are uniformly processed at the 3’end in a process known as cleavage and polyadenylation. Cleavage occurs at the poly(A) site, after recognition of the poly(A) signals in the pre-mRNA that are located in the 3′ untranslated regions (UTR) and the 3’flanking regions. Over 70% of mammalian genes undergo alternative polyadenylation (APA), where alternative poly(A) sites are utilised [3]. The regulatory powers of APA reside in the production of mRNA isoforms that differ in the lengths of their 3’UTRs. 3’UTRs harbour a plethora of regulatory elements that provide targets for RNA binding proteins or miRNAs which in turn can mediate stability, translatability or localisation of the respective transcript isoforms [4, 5]. Therefore, utilisation of alternative poly(A) sites through APA can post-transcriptionally regulate gene expression.

The relative frequencies between isoforms with long or short UTR length present in a cell represents the cellular APA profile. If cells enter a particular state or if they receive specific cues, the profiles can be changed to ensure adapted and adequate expression of the affected genes. Mechanistically, this can be achieved by favouring one poly(A) site over another at the point of co-transcriptional cleavage, known as active APA. The relative frequencies between the long and short isoforms in a cell can also be changed at the post-transcriptional level by selectively destabilising one isoform over another in the cytoplasm, known as passive APA [4].

Alterations in the transcriptome of patients with neurodegenerative diseases has been investigated with RNA-seq to assess changes in gene expression [6], splicing [7], and changes in miRNAs [8, 9] and lncRNAs expression [10]. However, there have been few studies that globally assess changes of APA profiles in neurodegenerative tissues or cells. Individual gene analysis has identified APA changes in genes associated with neurodegeneration, for example *MAPT* in AD [11, 12], *SNCA* in PD [13] and *TARDBP* in ALS [14]. However, to date transcriptome-wide APA profile changes have not been assessed for both AD and PD, and there has been only one such study focussing on ALS [15].

APA profiles are generally established using specific protocols that select and sequence only the very 3’ends of mRNAs (5). Recently a bioinformatics pipeline has been developed that enables APA profiles analysis from existing standard RNA-seq data sets [16]. This method, Dynamic Analysis of Alternative Polyadenylation from RNA-seq (DaPars), thus enables de novo identification and analysis of dynamic poly(A) site changes from any newly generated or deposited RNA-Seq data set.

The assessment of APA changes in the affected regions of neurodegenerative disease patients through wet-lab experiments is complicated by the scarcity of post-mortem patient RNA. However, using DaPars, APA can be investigated from standard RNA-seq data from existing studies. In this report, we use DaPars to compare APA profiles from sequenced RNA samples isolated from AD, PD and ALS patient with their respective control samples at a global scale. Using this approach, we identified individual genes that have previously been associated with the respective diseases and undergo disease specific APA changes. In addition, we find commonality between the diseases by showing that the genes which undergo disease-specific APA profile changes are associated with mitochondrial function and protein catabolism. As these processes are directly linked to neurodegeneration, our findings suggest that altered APA profiles may be a significant contributor to establish a transcriptome characteristic for a neurodegenerative state.

## Results

### APA in Alzheimer’s disease

Dysregulated RNA processing in AD has been identified in isolated cases, such as the extracellular aggregation of U1snRNP, a factor associated with regulation of splicing and polyadenylation, in AD brains [17]. Although certain genes associated with AD such as *COX-2* [18], *MAPT* [11, 12] and *APP* [19] utilise different 3’UTRs, there have been no genome-wide studies investigating the role of APA in AD.

To address this issue, we used an RNA-seq dataset from a study on transcriptomics of 4 Late Onset AD (LOAD) patients and 4 control individuals [10] and the DaPars pipeline to identify differences of UTR length in AD compared to healthy individuals. The RNA samples used had been deep sequenced on the Illumina platform to generate single end raw FASTQ files that were deposited on the Sequence Read Archive (GEO Accession number GSE24565). FASTQ files were groomed and aligned to the hg19 genome using TopHat using the Galaxy platform (https://usegalaxy.org/). The aligned BAM files were then converted to Bedgraph files for input into the DaPars script. Between 164.8–188.6 million reads per sample were subjected to DaPars analysis.

**Fig. 1:**
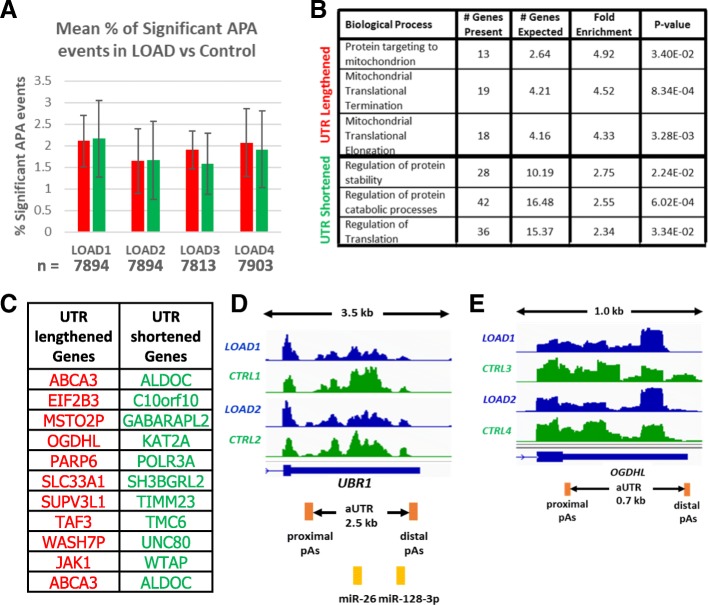
*UBR1* and *OGDHL* show significant APA changes in LOAD. **a** Bar chart showing mean percentage of significant 3’UTR lengthening (red bars) and 3’UTR shortening (green bars) events between each LOAD patient and control hippocampus derived RNA samples through DaPars analysis (PDUI > 0.25, Fisher’s exact Test, *p* < 0.05). The error bars indicate standard deviation from comparisons of 4 control samples. The mean number (n) of identified APA events for each LOAD patient are indicated underneath the chart. **b** Table showing the enrichment of biological processes identified with Gene Ontology Analysis using the PANTHER platform in genes exhibiting 3’UTR lengthening (upper three rows) and 3’UTR shortening (bottom three rows). **c** List of ten gene examples that show 3’UTR lengthening (red) or 3’UTR shortening (green) in LOAD samples compared to two or more control samples. **d** Genome browser view of *UBR1* as an example gene that shows 3’UTR shortening in LOAD (blue tracks) compared to control (CTRL, green tracks). Annotated miRNA target sites in the UBR1 3’UTR are indicated by yellow boxes below the gene structure. **e** Genome browser view of *OGDHL* showing 3’UTR lengthening in LOAD (blue tracks) compared to control (CTRL, green tracks). In **d** & **e**, the proximal and distal poly (A) sites are shown as orange boxes and the length of the alternative UTR (aUTR) is indicated below the gene structure. The length of the genome browser windows shown is indicated above in kilo bases (kb) between the two arrows

The RNA from this dataset originated from the hippocampus, the region of the brain important for memory, which is one of the first damaged regions in AD. As the patient and control samples were not accurately age-matched, each of the 4 LOAD patients were compared to each of four different control samples yielding a total of 16 comparisons. Between 7223 and 8419 APA events were identified and of those 0.5–3.3% of genes did undergo statistically significant APA changes when the LOAD and control samples were compared (Fig. 1a). Whilst we identified significant AD specific APA changes, no trend toward either 3’UTR shortening or lengthening was evident in the patient RNA samples.

We next interrogated the genes that showed altered UTR lengths in AD by subjecting them to a Gene Ontology (GO) analysis using the PANTHER Platform. This showed that the genes exhibiting 3’UTR lengthening in AD compared to healthy controls were enriched in mitochondrial pathways (Fig. 1b), while an enrichment in genes associated with protein catabolic processes was identified in those genes that had shorter 3’UTRs in AD derived samples. Interestingly, both biological processes identified by the GO analysis are closely linked to neurodegeneration. Therefore, changes in gene regulation through APA of these pathways may be a contributing factor to AD pathology.

We next isolated APA events that occurred in 2 or more LOAD patient samples compared to controls. Using this approach, we identified 10 genes that showed 3’UTR lengthening and 10 genes that showed 3’UTR shortening in 2 or more LOAD samples (Fig. 1c and Additional file 1: Figure S1). The ubiquitin ligase *UBR1,* is an example that showed 3’UTR shortening in AD in two patients versus control comparisons (Fig. 1d). Whilst UBR1, to the best of our knowledge, has not previously been directly implicated in AD, it is an E3 ubiquitin ligase of the N-end rule pathway [20] and mutations in the gene are linked to the Johanson-Blizzard syndrome which is characterised by a variety of features including varied degrees of cognitive impairment [21]. In addition, as protein turnover and degradation in general is associated with AD, altered expression of genes that are part of the UPP pathway such as *UBR1*, could contribute to dysregulation of protein turnover. Interestingly, the alternative long 3’UTR of the *UBR1* gene contains target sites for miR-26 and miR-128-3. MiR-26 has been shown to be upregulated in the temporal cortex of AD patients [22], while miR-128-3 was upregulated in the hippocampus of AD patients [23]. The upregulation of miR-26 and 128 in AD patients could result in the destabilisation of the longer *UBR1* UTR isoform and may thus provide a mechanistic explanation for the observed overrepresentation of transcripts with short 3’UTRs in AD patients.

Of the ten genes that showed 3’UTR lengthening in more than one LOAD-control comparison, *OGDHL* (Fig. 1e) is the most notable example. *OGDHL* encodes a brain- specific isoenzyme for oxoglutarate dehydrogenase that functions in the mitochondrial Krebs cycle. Downregulation of this protein, which may be aided by the UTR lengthening, has been observed in an AD mouse model [24] where its decreased expression can contribute to reduced ATP production.

We conclude from these results that whilst there is little overlap between APA events in LOAD and control comparisons, we nevertheless identified reoccurring APA events in a small number of genes that have previously been linked to neurodegeneration.

Although AD typically begins through degradation of the hippocampus in the temporal lobe region of the brain, other brain regions can be affected in AD [7]. We thus expanded our analysis of APA in AD by including an additional RNA-seq data set generated using RNA isolated from the frontal and temporal lobes of AD patients and control samples [7]. Notably, the read depth of this RNA-seq dataset was 10-fold lower than that of the previous dataset with just 13–15 million reads per sample. The samples were mapped and treated as described above. The samples were subsequently interrogated for significant UTR length changes by pumping them through the DaPars pipeline using the same parameters as described above.

**Fig. 2:**
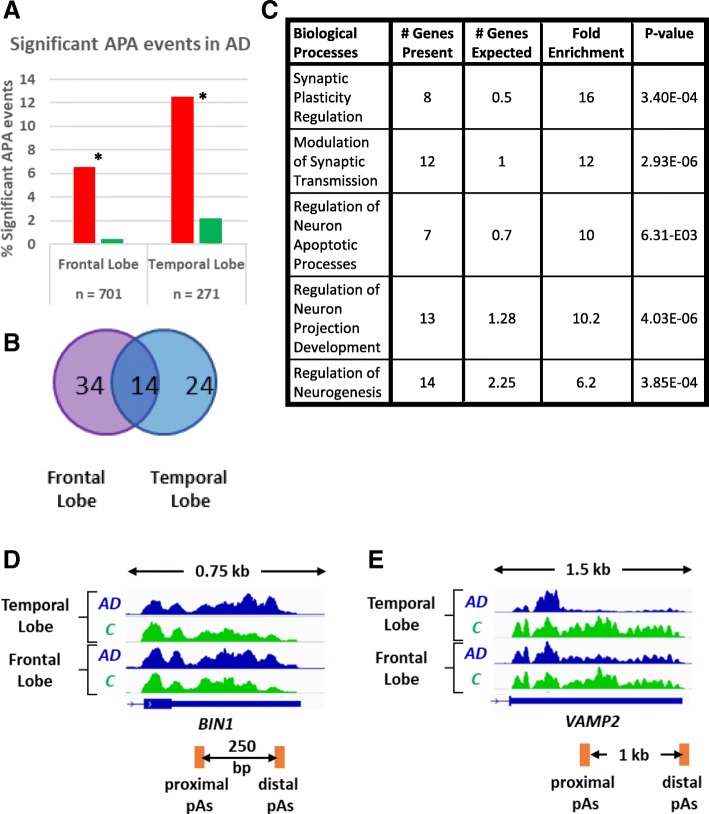
APA Regulation in Different Regions of the Brain in AD. **a** Significant 3’UTR lengthening (* = *p* < 0.01, Fisher’s exact Test) is observed in the frontal lobe and temporal lobe regions of the brain using DaPars analysis (PDUI > 0.25, Fisher’s exact Test < 0.05). Red and green bars indicate number of identified UTR-APA lengthening and shortening events respectively. The number of identified APA events in the comparison are indicated by n below the graph. **b** Venn diagram highlighting that different genes undergo APA in different regions of the brain and 14 genes show the same movements in both frontal (purple) and temporal lobe (blue). **c** Table to highlight the biological processes that were identified through Gene Ontology Analysis using the PANTHER platform in the genes that showed 3’UTR lengthening in either cohort. **d** Genome browser view of the 3’UTR lengthening in *BIN1* in AD (AD, blue tracks) compared to control (C, green tracks). **e.** 3’UTR shortening in *VAMP2* in AD (blue tracks) compared to control (C, green tracks). In **d** & **e**, the proximal and distal PAS’s are shown as orange boxes and the length of the alternative UTR (aUTR) is indicated below the gene structure. The length of the genome browser windows shown is indicated above in kilo bases (kb) between the two arrows

The comparison of APA using RNA extracted from the Frontal Lobe identified 701 genes that showed changes in UTR length in AD compared to the control samples and 7.0% of these were statistically significant. Comparisons of RNA from the temporal Lobe in this data set identified 271 APA events of which 14.7% were statistically significant. Unlike in the previous data set, the genes that did show significant APA, tended to lengthen their 3’UTRs in both the temporal and frontal lobes (Fig. 2a). However, the overlap of genes showing significant APA in both brain regions was limited to 14 genes (Fig. 2b). This indicates that APA is differentially regulated between the temporal and frontal lobes in AD.

To characterise the cohort of genes that show AD patient-specific UTR lengthening further, we subjected them to a GO analysis using the PANTHER platform, which showed an enrichment of synaptic plasticity regulation, modulation of synaptic transmission and regulation of neuron apoptotic processes (Fig. 2c). This suggests that APA mediated regulation of the transcriptome could affect synaptic activity, which may be a contributing factor for altered neuronal function as observed in AD. A notable gene that undergoes lengthening in both the frontal and temporal lobes of AD patients compared to the controls is *BIN1*. The *BIN1* gene, whose protein is involved in synaptic vesicle endocytosis, showed 3’UTR lengthening in the temporal and frontal lobe samples but not in the whole brain sample, qualifying it as a localised APA change (Fig. 2d). Importantly, this gene has been identified as a risk locus for AD [25] and increased expression has been linked to modulating tau pathology in LOAD [26]. The lengthening of the 3’UTR may contribute to increased expression of BIN1 protein and could so be a contributing factor for AD.

We identified six genes that showed 3’UTR shortening, and only one of these, *VAMP2,* showed APA in both the frontal and temporal lobe region of the brain (Fig. 2e). However, *VAMP2* encodes a protein that is involved in neurotransmitter release during the fusion of synaptic vesicles to the pre-synaptic membrane. Most interestingly, decreased protein expression of VAMP2 is seen during progression of AD [27], which may contribute to AD pathology.

These results show that different genes exhibit significant APA in different regions of the AD brain, but all show a significant trend to 3’UTR lengthening. Altered protein expression of BIN1 and VAMP2 have been associated with AD [26, 27]. In some patients, this may be at least partially caused by APA. Therefore, our analysis suggests a novel mechanism of *BIN1* and *VAMP2* gene regulation that may be disrupted in AD patients.

### APA in Parkinson’s disease

Parkinson’s Disease (PD) is characterised by bradykinesia, tremors and stiffness of movement, due to a loss of dopaminergic neurons in the substantia nigra region of the brain. These neurons use dopamine as their neurotransmitter, which has reduced levels in PD. Further, genes involved in dopamine metabolism show altered expression in PD [28]. PD is also characterised by the accumulation of cytoplasmic protein aggregates, mostly composed of insoluble α-synuclein forming structures called Lewy bodies [29]. Although 90% of PD cases are sporadic, much of the research has been focussed on familial cases of the disease. Sporadic PD (S-PD) patients exhibit the same symptoms, and in some cases, mutations have been seen in the same causative genes as in familial PD (F-PD). These genes have been studied extensively (Reviewed in [28, 30]).

To date, only one study has assessed the role of APA in PD. Rhinn and colleagues showed an increased usage of the distal poly(A) site of *SNCA*, a gene crucial to PD pathogenesis, in the brains of PD patients. These longer isoforms produced proteins that were localised to the mitochondria and were more likely to aggregate suggesting a critical role of APA in α-synuclein regulation [13].

To assess APA changes in S-PD patients, an RNA-seq dataset from three S-PD and control patients was subjected to DaPars analysis. The RNA had been isolated from midbrain dopaminergic neurons derived from S-PD patient iPSCs. These dopaminergic neurons showed signs of oxidative stress and altered neuronal activity, as observed in the PD disease state. The paired-end FASTQ files were groomed and mapped to hg19 using TopHat on the Galaxy platform, resulting in 49.4–54.8 million reads per sample.

**Fig. 3:**
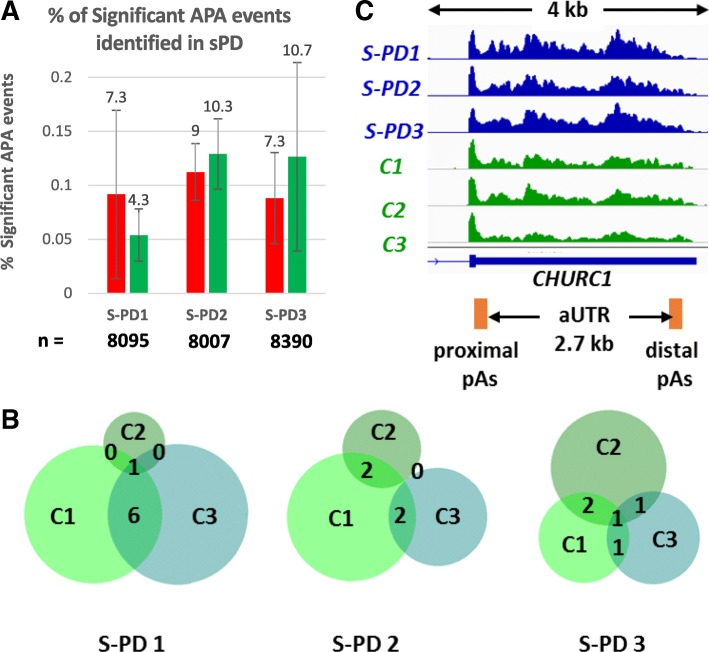
APA Regulation in sPD. **a** Bar chart showing the mean percentage of 3’UTR lengthening (red) and shortening (green) APA events in SP-D compared to each of the three control samples identified with DaPars analysis (PDUI > 0.25, Fisher’s exact Test *p* < 0.05). Error bars show standard deviation between the 3 comparisons. The mean number of APA events identified for each comparison is indicated by n below the chart. The percent of statistically significant APA events are labelled. **b** Venn diagrams showing the overlaps between the identified APA events for each of the S-PD vs control samples. Each shade of green represents a comparison to a different control sample C1, C2 or C3. **c** Genome browser view depicting the 3’UTR lengthening in *CHURC1* gene that shows lengthening in each S-PD sample (blue tracks) compared to control samples (C, green tracks)*.* The proximal and distal poly(A) sites are shown as orange boxes and the length of the alternative UTR (aUTR) is indicated below the gene structure. The length of the genome browser windows shown is indicated above in kilo bases (kb) between the two arrows

APA analysis was conducted on combined biological replicates of each of the three S-PD patient with three control RNA samples from derived dopaminergic neurons. The biological replicate bedgraph files were combined and DaPars analysis was conducted on the mean of the reads from the two replicates. The UTR length changes in each patient sample was compared with each control sample and the mean of the total number of APA events per S-PD vs control comparison was calculated. Less than 0.2% of genes were identified to show significant APA changes between patient and control samples (Fig. 3a). This suggests that APA is unlikely to be a major contributor to transcriptome changes that have been observed in S-PD [31]. Furthermore, we observed great variation between the number of APA events identified between the different comparisons as highlighted by the standard deviation and there was very little overlap between the genes exhibiting APA between different comparisons (Fig. 3b). Taken together, these results suggest that few APA changes can be specifically correlated to S-PD.

An exception to the above statement is the *CHURC1* gene which showed 3’UTR lengthening in each of the S-PD patients compared to at least 2 of the control samples (Fig. 3c). Interestingly, *CHURC1* encodes a transcriptional activator that is involved in neuronal development, but it has, to the best of our knowledge, so far not been associated with neurodegeneration.

Transcriptomic changes between familial (F-PD) and sporadic PD (S-PD) cases have also been assessed through RNA-seq [32]. In this study, induced pluripotent cells (iPSCs) were derived and differentiated to midbrain dopaminergic neurons from fibroblasts isolated from a familial PD patient with the N370S mutation in the *GBA* gene, an S-PD patient and two healthy controls. We used DaPars on this RNA-seq dataset to evaluate potential UTR length changes in PD in the familial and sporadic patients compared to the healthy individuals. Biological replicates each having between 25.8–37.2 million reads, were subjected to DaPars analysis as described above.

**Fig. 4:**
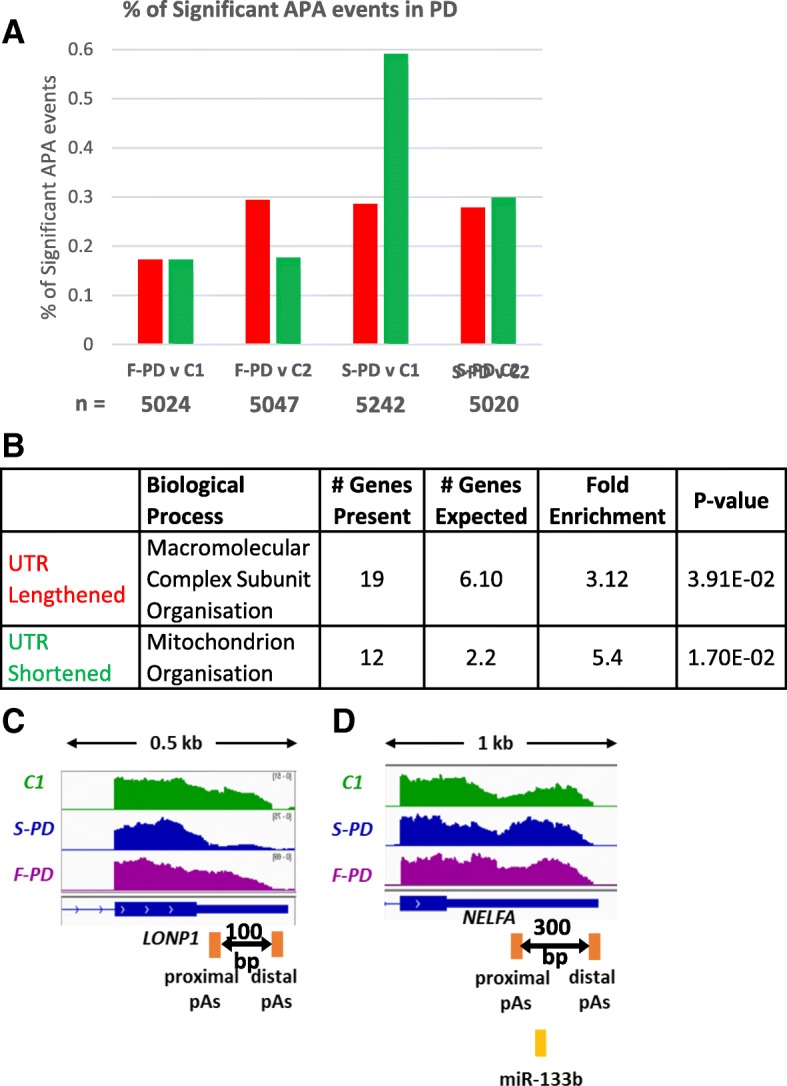
APA Regulation in F-PD compared to S-PD. **a** Bar chart to show the percentage of 3’UTR lengthening (red) and 3’UTR shortening (green) events in Familial-PD (F-PD) and Sporadic-PD (S-PD) compared to the two healthy controls (C1, C2) identified by DaPars analysis (PDUI > 0.25, Fisher’s exact Test *p* < 0.05). The total number of identified APA events are indicated by n beneath the chart. **b** A table showing the biological processes identified through GO analysis on the PANTHER platform for those genes that undergo significant APA either in S-PD an or F-PD. **c** Genome browser view of *LONP1* for which 3’UTR shortening was observed in S-PD (blue track) but not F-PD (purple track) when compared to the control (green track). **d** Genome browser view of the 3’UTR lengthening observed in *NELFA* in F-PD (F-PD, purple track) compared to control (C, green track) but not in S-PD (S-PD, blue track). **c** & **d**, the proximal and distal poly(A) sites are shown as orange boxes and the length of the alternative UTR (aUTR) is indicated below the gene structure. The length of the genome browser windows shown is indicated above in kilo bases (kb) between the two arrows

There was no clear trend to toward either 3’UTR shortening or lengthening apparent and overall fewer than 0.6% of APA events proved significant (Fig. 4a), again indicating that global APA changes do not occur in PD. However, GO analysis of all of the genes that show 3’UTR shortening presented an enrichment in mitochondrial organisation (Fig. 4b). Altered regulation of these genes through shorter 3’UTRs may have a role in mediating mitochondrial dysfunction, which is an established phenotype in PD. Genes showing 3’UTR lengthening were associated with ‘macromolecular complex subunit organisation’, including genes associated with protein turnover (Fig. 4b). Aggregation of misfolded proteins is an important hallmark of PD pathogenesis, therefore altered regulation of these genes through 3’UTR lengthening may contribute to PD pathology.

A prominent candidate gene showing 3’UTR shortening in S-PD, but not F-PD, is *LONP1* (Fig. 4c), which encodes a mitochondrial matrix protease that mediates degradation of misfolded or oxidatively damaged proteins. Given the association of misfolded protein aggregation in PD, including in the mitochondria [33], alterations in the regulation of the *LONP1* could affect mitochondrial function. In particular, *LONP1* knockdown has been shown to cause an increase in PINK1 accumulation, which increased mitophagy [34]. PINK1 is associated with F-PD along with Parkin, which together regulate the removal of dysfunctional mitochondria. Consequently, alterations in LONP1 protein levels in the mitochondrial matrix, may contribute to PD pathogenesis. The mechanisms by which LONP1 3’UTR length is dysregulated in PD remains unclear and no RBP sites or regulatory elements in the alternative UTR (aUTR) that could influence transcript stability, protein output or localization were not identified by scanning these sequences.

An example of F-PD specific 3’UTR lengthening was seen in *NELFA* (Fig. 4d). *NELFA* encodes a component of the negative elongation factor complex, which has a role in transcriptional pausing and regulating Pol II mediated transcription. A target site for miR-133 family of miRNAs is found in the aUTR which may mediate the isoform expression in the cytoplasm. miR-133b was shown to be deficient in the midbrain of PD patients [35], which could explain this 3’UTR lengthening event.

These results indicate APA is not globally deregulated in either S-PD or F-PD compared to their respective controls. However, the genes that do undergo APA in S-PD or F-PD are associated with mitochondrial function and protein degradation suggesting that a potential role of APA in PD pathology.

### APA in ALS

Amyotrophic Lateral Sclerosis (ALS), also known as Motor Neuron Disease, is a debilitating disease which results in death of upper and lower motor neurons which control voluntary muscles, leading to muscle stiffness, weakness and eventually loss of voluntary movement. Like AD and PD over 90% of cases are sporadic, while approximately 10% are related to inherited genetic mutations. However both familial (fALS) and sporadic (sALS) cases exhibit similar neuropathologies both fALS and sALS patient samples have shown similar dysregulation of miRNA and gene expression [9].

ALS has been closely linked to defects in RNA metabolism. Mutations in the nuclear localisation sequence (NLS) of *FUS* and *TARDBP*, two genes that encode RBPs associated with RNA processing, are linked to fALS. These mutant proteins subsequently aggregate in the cytoplasm forming inclusion bodies which contribute to loss of motor neuron function [36]. FUS, which has a role in alternative splicing [37], can also interact with the Pol II CTD to regulate phosphorylation of Ser2 [38] and can control mRNA turnover [39]. Furthermore, FUS knockdown altered mRNA expression of genes associated with mitochondrial function and increased proximal poly(A) site usage [38]. More recently, FUS was shown to affect poly(A) site usage depending on the proximity of its binding site to the poly(A) site [40]. FUS therefore functions in many aspects of mRNA regulation. TDP-43, which is encoded by the *TARDBP* gene, was shown to autoregulate the splicing and poly(A) site selection of its own transcript [14], although its role in global poly(A) site choice has not been investigated. Thus, the regulation of RNA metabolism by these RBPs is crucial in ALS. Both FUS and TDP-43 regulate alternative splicing of genes involved in neuronal development and neurodegeneration, but there is not a significant overlap in the genes they regulate [41].

Changes in the 3’UTR length in both sALS and a *C9orf27* mutant case of ALS have been assessed globally through RNA-seq [15]. APA was shown to be altered differentially in these two patients compared to the healthy control, and different regions of the brain showed widespread different poly(A) site usage [15]. While these regions of the brain are important in ALS, it is the motor neurons whose function is mainly compromised. Here, APA changes in ALS were investigated with DaPars on an RNA-seq dataset where RNA had been isolated from motor neurons of the lumbar spinal cord which had not been fully degenerated from ALS patients [42].

**Fig. 5:**
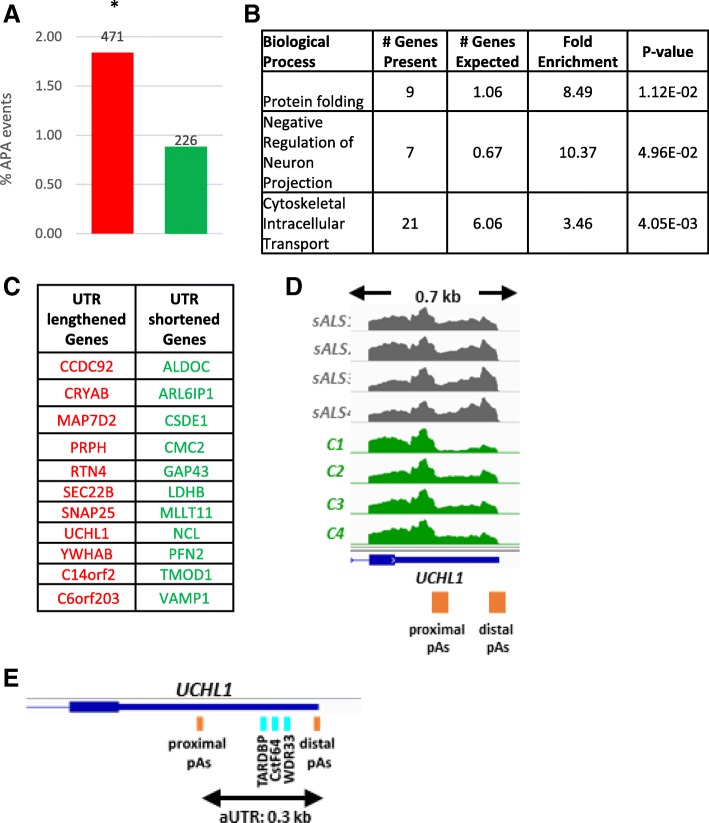
APA Regulation in Motor Neurons in sALS. **a** Bar chart showing the percentage of genes that showed significant UTR changes through DaPars analysis (PDUI > 0.25, Fisher’s exact test, *p* < 0.05). In total, 1.8% of genes showed 3’UTR lengthening (red), while fewer than 1% showed 3’UTR shortening (green) in all comparisons between 10 sALS and 8 control samples, showing a significant trend to 3’UTR lengthening (* = *p* < 0.05, Fisher’s exact Test). The data labels indicate number of APA events identified among all cross comparisons. **b** Table highlighting the biological processes that are enriched in the genes showing UTR lengthening identified with GO analysis using the PANTHER platform. The cohort of genes showing UTR shortening were not enriched in any biological processes. **c** Table of 22 genes showing 3’UTR lengthening or shortening events and occur in at least 3 different sALS vs control comparisons. **d** Genome browser view of the 3’UTR lengthening seen in *UCHL1* gene in sALS (grey tracks) compared to control (green tracks). The proximal and distal poly(A) sites are shown as orange boxes the length of the genome browser window shown is indicated above in kilo bases (kb) between the two arrows. **e** Schematic indicating the position of RBPs found uniquely in the aUTR of *UCHL1* are indicated by aqua boxes. The length of the alternative UTR (aUTR) is shown between the arrows below the gene structure

To assess whether APA is deregulated in ALS we considered deposited RNA-seq data from 13 sALS and 9 control individuals for DaPars analysis. Samples with fewer than 20 million reads, which fall below the 50 million reads threshold requirements for DaPars were omitted, yielding a final 10 sALS and 8 control patients. Each of the selected sALS patient samples were compared to each of the controls, resulting in 80 comparisons. For each patient and control comparison, on average, 2.7% of genes showed significant APA, with twice as many 3’UTR lengthening than shortening events (Fig. 5a). GO analysis for UTR lengthened genes identified an enrichment for genes involved in negative regulation of neuron projection development as well as genes associated with cytoskeleton intracellular transport (Fig. 5b). Therefore, although global changes are not seen in sALS, APA may be important in the regulation of transcripts encoding proteins that contribute to neuropathology.

To investigate the key changing genes further, 3’UTR shortening or lengthening events occurring in at least 3 different sALS vs control comparisons were selected, yielding 22 genes (Fig. 5c). Of this cohort only one gene has previously been linked to neurodegenerative diseases, *UCHL1*. *UCHL1*, which encodes an abundant neuron specific enzyme associated with the UPP, shows 3’UTR lengthening in 4 sALS compared to 4 different control samples (Fig. 5d). UCHL1 is a regulator of ubiquitin turnover as it has both hydrolase activity to remove ubiquitin [43] and ubiquitin ligase activity [44]. Its crucial role in maintaining healthy motor neurons was demonstrated in a mouse knockout model where loss of UCHL1 increased ER stress causing motor neuron degeneration [45]. Furthermore, reduced UCHL1 protein levels [46], and loss of its activity have been reported in PD and AD [47].

Analysis of the *UCHL1* UTR using a CLIP database (http://lulab.life.tsinghua.edu.cn/clipdb/) identified 21 RBPs that can interact with this UTR, but only 3 of these, CstF-64, WDR33 and TARDBP, uniquely interact with the aUTR (Fig. 5e). CstF-64 promotes usage of a proximal poly(A) site, while WDR33, though crucial for cleavage and polyadenylation, does not have an established role in APA. Mutations in *TARDBP*, which mediates splicing, are linked to fALS cases but there is no evidence that it influences APA in genes apart from its own transcript [14]. Further, no miRNAs have been identified to target the *UCHL1* UTR. It is therefore unclear how the 3’UTR lengthening event in *UCHL1* may arise in sALS, yet dysregulation of this gene by APA may affect its protein output or localisation, which in turn could contribute to pathology in sALS.

Overall, few significant APA events were identified in sALS patients compared to the frontal cortex and cerebellum of the brain as were reported by Prudencio and colleagues. However, 3’UTR lengthening was identified in a number of genes including *UCHL1*, which is important in the UPP pathway and is associated with neurodegeneration.

## Discussion

The usage of alternative splicing and alternative promoter usage has been implicated in the brain to achieve the complexity required [7]. APA has also been investigated, with UTRs globally appearing to be lengthened in the brain compared to other tissues [48]. However, the possibility of changes in poly(A) site usage in neurodegeneration has not been extensively investigated at a global level.

A number of methods and pipelines have been developed to analyse APA that rely on the sequencing of the very 3’end of mRNAs [49]. As these approaches generally require tailor- made sequencing libraries, they are less suitable for the analysis of standard RNAseq data. To address this shortcoming several approaches have been developed [50–52] including DaPars [16]. DaPars is an established method and uses a sophisticated algorithm that uses a regression model for the identification of the distal and predictive proximal poly(A) sites in genes from standard RNA seq data sets. Importantly, this bioinformatics method has been successfully used to investigate APA from RNAseq data associated with a number of diseases [15, 16, 53, 54].

Here, RNA-seq datasets from AD, PD, and ALS were investigated with DaPars to assess alterations in APA (Additional file 1: Table S1). While widespread changes in APA were not seen in any of the diseases, examples of genes which may contribute to the disease state were identified. However, we cannot rule out that the lack of identifying prevalent disease associated changes in APA may have been limited by the small sample sizes used in this analysis. Furthermore, different APA profiles were observed in different regions of the brain, and whilst distinctly different genes were subjected to APA in the three different neurodegenerative diseases they encoded proteins functionally associated with mitochondrial function band protein catabolism.

### APA in AD

Assessment of APA in the two AD datasets investigated identified different cohorts of genes regulated by APA, however in each case, 3’UTR length varied in genes encoding proteins associated with AD pathology.

In the first AD dataset, RNA isolated from the hippocampal region between LOAD patients and controls was investigated for APA changes. A large degree of variation in APA was observed between different patient and control comparisons, suggesting that there is either natural variation between samples, or changes that are of collateral nature and thus may not be due or unique to AD. Here, only genes which showed APA in at least 3 of the controls were selected for further analysis eliminating more than 80% of the identified APA events. This stringent cut off makes the APA event more likely to be linked to AD instead of other unknown factors. However, it is important to keep in mind that although similar phenotypes are seen in different AD patients, AD is a composite disease, with varying pathologies associated to each case. Therefore, the same genes may not be affected by APA in each AD sample.

The RNA-seq dataset we used was originally investigated for transcriptome changes between LOAD and control samples and identified novel lncRNAs which were proposed to contribute to AD pathology [10]. Changes in gene expression in 113 protein coding genes were recognised [10], but none of these genes were identified as showing APA in our DaPars analysis, suggesting APA does not affect the stability of the resulting APA mRNA isoforms in AD but instead may alter protein production or localisation.

With the second dataset we assessed APA changes in the temporal and frontal lobe of the brain [7]. While we observed 3’UTR lengthening in both brain regions, most APA events were region-specific. In addition, we identified little overlap between genes that are subjected to APA in the lobes and the hippocampus (Additional file 1: Figure S2), which again suggests that these different brain regions have distinct APA profiles.

In the AD brain, it has previously been shown that genes associated with neuron structure and synapse function have altered expression [7]. Interestingly, our analysis identified genes associated with synaptic function and show significant APA profile changes between control and AD; as exemplified by *BIN1* and *VAMP2*. Whether the APA changes observed in this gene cohort are physiologically relevant is unclear. However, it is well established that APA can affect protein output and localisation [4] and changing protein expression of BIN1 [26] and VAMP2 [27] have previously been associated with AD. It is therefore plausible that 3’UTR lengths changes of *BIN1* and *VAMP2* in AD may impact on the final protein output of these genes which in turn may contribute to the disease state or progression.

Changes in splicing patterns have been linked to age in humans [55] and reduced expression of the nervous system specific RBP Nova1/Nova2 was linked to altered splicing in AD patients, but not cognitively normal aged patients [55]. Nova2 can also modulate poly(A) site choice by binding to a YCAY cis-element which inhibits co-transcriptional usage of a proximal poly(A) site through steric hindrance [56]. Decreased Nova expression may result in 3’UTR shortening in AD, but this was not observed on a global scale in any of these AD datasets. However, Nova binding sites were identified in the aUTR of *UBR1* and *VAMP2* [57], which both showed 3’UTR shortening in AD. Further functional studies will be required to investigate if reduced Nova expression affects poly(A) site choice of these transcripts. RBPs are important in regulating APA, and their function has been shown to be dysregulated in disease [58]. Altered availability of RBP binding sites through changes in 3’UTR length could thus play a significant role in the deregulation of genes in AD.

Defects in RNA metabolism have been implied in AD [17], and this analysis gives an indication that APA in relevant genes may also be dysregulated. However, genes known to be directly associated with AD, such as *PS1, PS2, APP* and *APOE*, did not show changes in poly(A) site usage in any of the datasets we interrogated, suggesting no role of APA in their regulation. However, with *UBR1* and *VAMP2* we did identify APA changes in physiologically relevant genes which could contribute to AD pathology.

### APA in PD

There is evidence that APA occurs in PD associated genes, such as α-synuclein [13], however changes in APA in PD have not been assessed on a global level. Here, RNA-seq data from patient and control iPSCs differentiated to dopaminergic neurons from two different datasets was used to assess 3’UTR length changes in PD compared to controls.

The first dataset assessed APA in dopaminergic neurons derived from iPSCs in 3 S-PD patients. Far fewer APA events were identified compared to control samples, suggesting APA changes are unlikely to be a major causative or consequential of the PD phenotype.

Comparison between F-PD and S-PD RNA-seq data with control individuals in the second RNA-seq dataset did again not show global changes in APA. However, the relative small number of genes that show altered 3’UTR length in PD versus controls, were associated with mitochondrial organisation or alterations in macromolecular subunit organisation, i.e. protein aggregation. These are two key phenotypes seen in PD, suggesting that APA may be affecting individual genes that are directly related to the disease state.

While, evidence of APA in α-synuclein has been described in PD to influence transcript localisation, and therefore protein localisation [13], no significant differences in 3’UTR length were seen in either S-PD or F-PD (Additional file 1: Figure S3), suggesting in this case, APA does not contribute to dysregulated α-synuclein expression in PD.

In conclusion, our data suggest that whilst APA may not have a global impact on gene expression changes in PD, it nevertheless may regulate the expression of a select few physiologically relevant genes including *LONP1* and *NELFA* that could contribute to PD pathology.

### APA in ALS

Defects in RNA processing have been linked to ALS, primarily due to mutations in FUS and TDP-43 [37]. In addition, depletion of *HNRNPA2B1*, mutations in which are associated with ALS, was shown to promote usage of distal poly(A) sites [59].

APA changes using DaPars in ALS have previously been explored and more APA changes were observed in sALS compared to c9ALS [15]. This genome-wide study by Prudencio and colleagues assessed transcriptome-wide changes in the cerebellum and frontal cortex which are not the focal points of degeneration in ALS, although these regions may have compromised function [60]. To further investigate APA in ALS, we used a publicly available RNA-seq dataset from spinal cord motor neurons, which is a physiologically highly relevant region for ALS. We found that around 700 genes (2.7%) of genes showed significant APA changes, and an overall trend to 3’UTR lengthening was observed in patient derived RNA. Whether any of these UTR lengthening events are physiologically relevant is unclear but the affected genes are enriched for GO terms such as neuron projection development and cytoskeleton intracellular transport. Furthermore, the 3’UTR lengthening event identified in the *UCHL1* gene is of particular interest as the lengthening was observed in three of the sALS versus control comparisons. Most interestingly, reduced UCHL1 protein expression has been linked to neurodegeneration [46], and the loss of this enzyme contributes to motor neuron degeneration [45].

## Conclusions

This paper assesses APA changes in different neurodegeneration diseases using existing publicly available RNA-seq datasets. Although widespread APA changes were not observed, for each neurodegenerative disease we identified APA events that occur in physiologically relevant genes. In particular we identified *UCHL1, BIN1* and *VAMP2* as examples that show disease specific UTR lengths changes that may contribute to the pathology of the respective diseases. Furthermore, we show that whilst different genes do show APA in AD, PD and ALS, in all three pathologies genes encoding proteins associated with the UPP were overrepresented indicating that deregulation of such genes by APA may be a common pathology. Finally, there is increasing evidence showing that changes in RNA metabolism, including in splicing [17] are associated with neurodegenerative disease states, to which APA can be now added.

## Methods

Raw sequencing data in the form of FASTQ files and alignment information was downloaded from the Sequence Read Archive (SRA). GEO Accession numbers of datasets used are outlined in Table 1. FASTQ files were groomed and filtered reads were aligned to the human genome (hg19) using TopHat using Galaxy (https://usegalaxy.org/). The aligned BAM files were converted to bedgraph files and subjected to Dynamic Analysis of Alternative Polyadenylation from RNA-Seq (DaPars) [16, 61]. The code is available at https://github.com/ZhengXia/DaPars. This python script enables de novo identification and analysis of dynamic poly(A) sites from RNA-Seq data. In DaPars, the distal poly(A) site is considered to be the default poly(A) site in the transcript, and de novo poly(A) sites are identified using a linear regression model. The read intensities are modelled as a linear combination of proximal and distal poly(A) sites at a single nucleotide resolution. The relative usage of proximal and distal poly(A) sites can be assessed by looking at the percentage of reads which use the distal instead of the proximal poly(A) site (percentage of distal poly(A) site usage index, PDUI). The PDUI is calculated in multiple data sets and the difference in PDUI between different data sets is calculated. Positive or negative PDUIs determine whether a statistically significant APA event is identified as 3’UTR lengthening or shortening respectively between two samples [16]. Genes with more than a 25% change between patient and control are highlighted as being statistically significant changes in poly(A) site usage. The output from DaPars was coupled to known poly(A) site coordinates ensuring false poly(A) sites are not identified. An additional filter in the DaPars script was added so that the predicted proximal poly(A) site was present within 250 nucleotides of a previously annotated poly(A) site, rather than 500 nucleotides as initially proposed by the authors [61]. Bedgraph files were converted to bigwig files for visualisation of the 3’UTRs on the IGV Browser.

**Table 1:** GEO Accession Numbers of publicly available RNA-seq data

GEO/SRA Accession Number	Background to Dataset	Reference
GSE67333	RNA sequencing on RNA samples extracted from the hippocampi of 4 LOAD patients and 4 age-matched controls	[10]
SRX034874	Total mRNA from Capital Biosciences sequenced from Total Brain, Frontal Lobe and Temporal Lobe from Normal individuals and AD patients	[7]
GSE62642	Total RNA extracted from dopaminergic neurons derived from iPSCs from healthy controls, sporadic PD patient, and monozygotic twins where one individual has PD and one does not. Two technical repeats per sample	[32]
ERA589991	Total RNA extracted from dopaminergic neurons derived from iPSCs of sporadic PD patients and healthy individuals. Two technical replicates per sample.	N/A
GSE76220	Total RNA-sequencing on RNA from Motor Neuron Populations Isolated from sALS	[42]

## Additional file

Additional file 1: **Figure S1.** APA Heat map for the ten genes that undergo shortening of their 3’UTRs and ten genes that show 3’UTR lengthening in LOAD samples compared to two or more control samples. The gene names are indicated on the left (10 lengthening genes and ten shortening genes as per Fig. 2c) The percentage of distal poly(A) site usage index values (PDUI) range from shades of green indicating shortening and shades of red indicating Lengthening. The different comparisons between diseased (AD1–4) and controls (C1-C4) are outlined on the X-axis. **Figure S2.** Different genes affected by APA in different regions of the brain. **A.** Venn Diagram to show the overlap of genes regulated by APA in the hippocampus from the LOAD Dataset (teal) and the frontal and temporal brain region dataset (blue). **B.** Table to show the APA change of the 21 genes identified to show altered UTR lengths with DaPars analysis (PDUI > 0.25, Fisher’s exact Test, *p* < 0.05) in the two AD datasets assessed. The genes that showed 3’UTR lengthening or 3’UTR shortening in both datasets have been separated from those that showed differential APA regulation. **Figure S3.** *SNCA* does not show UTR length changes in PD. **A.** Genome browser view of *SNCA* in the first PD dataset assessed to show no change in UTR length in the three S-PD samples (blue tracks) compared to control samples (green tracks). **B.** Genome browser view of *SNCA* in the second PD dataset assessed to show no change in UTR length in S-PD (blue track) or F-PD (purple track) compared to control (green tracks). In **A** & **B,** the length of the genome browser window shown is indicated above in kilo bases (kb) between the two arrows. **Table S1.** Summary of all the data sets used in Figs. 1, 2, 3 4, 5. Details regarding the data sets used to in the analysis’ that lead to the data presented in Figs. 1, 2, 3 4, 5 are given. (PPTX 342 kb)

## Abbreviations

3’UTR: 3’Untranslated Region
AD: Alzheimer’s Disease
ALS: Amyotrophic Lateral Sclerosis
APA: Alternative Polyadenylation
DaPars: Dynamic analysis of Alternative Polyadenylation from RNA-Seq
EOAD: Early Onset Alzheimer’s Disease
GO: Gene Ontology
LOAD: Late Onset Alzheimer’s Disease
NLS: Nuclear Localisation Sequence
PD: Parkinson’s Disease
poly(A): polyadenylation
RNA: Ribonucleic acid
