# Supplementary material for: Alternative cleavage and polyadenylation of genes associated with protein turnover and mitochondrial function are deregulated in Parkinson’s, Alzheimer’s and ALS disease
Source: BMC Med Genomics. 2019 May 9;12:60. doi: 10.1186/s12920-019-0509-4 (PMC6507032; doi:10.1186/s12920-019-0509-4)
Supplement: Supplementary file 1 — Figure S1. APA Heat map for the ten genes that undergo shortening of their 3’UTRs and ten genes that show 3’UTR lengthening in LOAD samples compared to two or more control samples. The gene names are indicated on the left (10 lengthening genes and ten shortening genes as per Fig. 2c) The percentage of distal poly(A) site usage index values (PDUI) range from shades of green indicating shortening and shades of red indicating Lengthening. The different comparisons between diseased (AD1–4) and controls (C1-C4) are outlined on the X-axis. Figure S2. Different genes affected by APA in different regions of the brain. A. Venn Diagram to show the overlap of genes regulated by APA in the hippocampus from the LOAD Dataset (teal) and the frontal and temporal brain region dataset (blue). B. Table to show the APA change of the 21 genes identified to show altered UTR lengths with DaPars analysis (PDUI > 0.25, Fisher’s exact Test, p < 0.05) in the two AD datasets assessed. The genes that showed 3’UTR lengthening or 3’UTR shortening in both datasets have been separated from those that showed differential APA regulation. Figure S3. SNCA does not show UTR length changes in PD. A. Genome browser view of SNCA in the first PD dataset assessed to show no change in UTR length in the three S-PD samples (blue tracks) compared to control samples (green tracks). B. Genome browser view of SNCA in the second PD dataset assessed to show no change in UTR length in S-PD (blue track) or F-PD (purple track) compared to control (green tracks). In A & B, the length of the genome browser window shown is indicated above in kilo bases (kb) between the two arrows. Table S1. Summary of all the data sets used in Figs. 1, 2, 3 4, 5. Details regarding the data sets used to in the analysis’ that lead to the data presented in Figs. 1, 2, 3 4, 5 are given. (PPTX 342 kb) [file 12920_2019_509_MOESM1_ESM.pptx]

## Slide 1
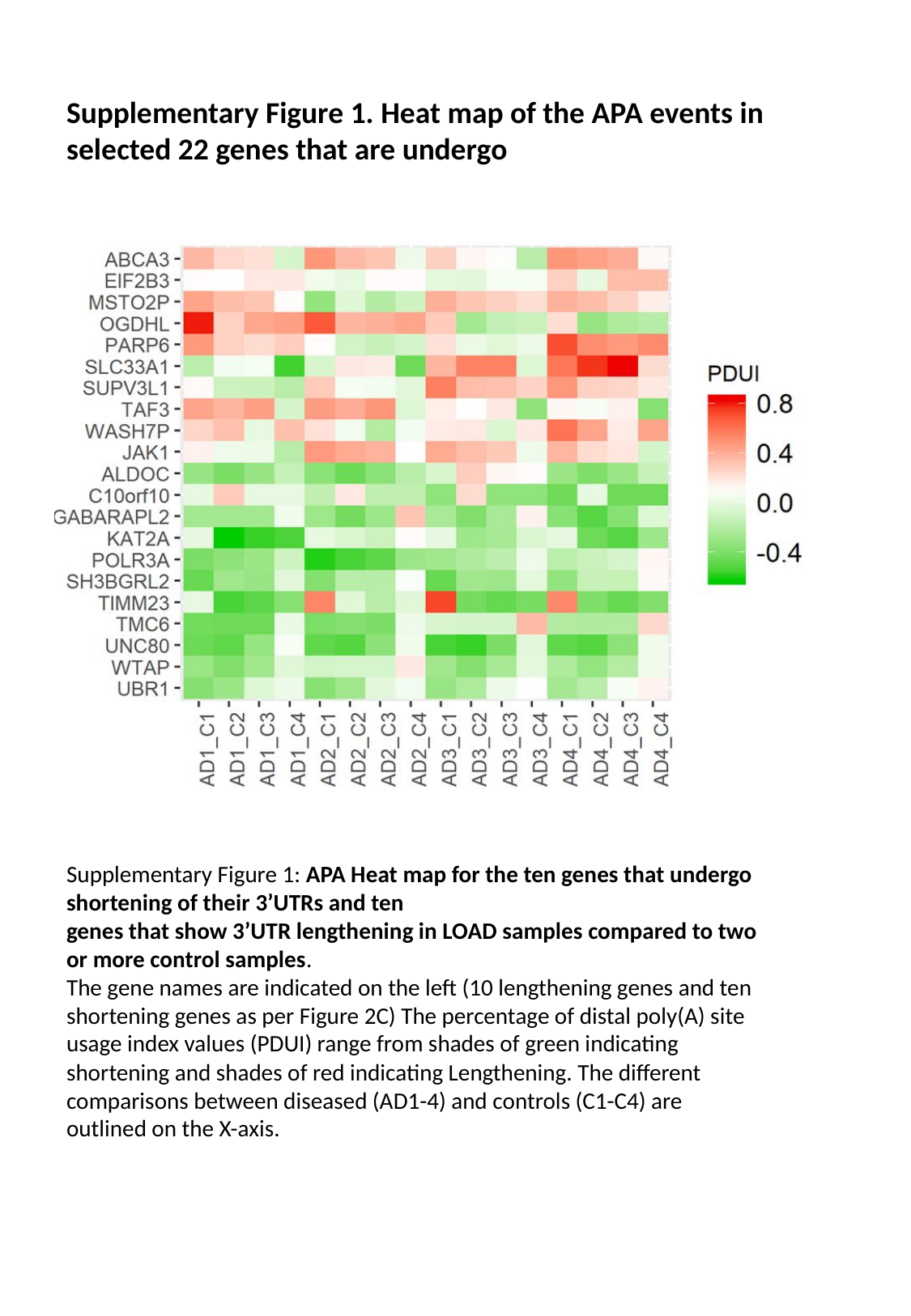

Supplementary Figure 1. Heat map of the APA events in selected 22 genes that are undergo
Supplementary Figure 1: APA Heat map for the ten genes that undergo shortening of their 3’UTRs and ten
genes that show 3’UTR lengthening in LOAD samples compared to two or more control samples.
The gene names are indicated on the left (10 lengthening genes and ten shortening genes as per Figure 2C) The percentage of distal poly(A) site usage index values (PDUI) range from shades of green indicating shortening and shades of red indicating Lengthening. The different comparisons between diseased (AD1-4) and controls (C1-C4) are outlined on the X-axis.

## Slide 2
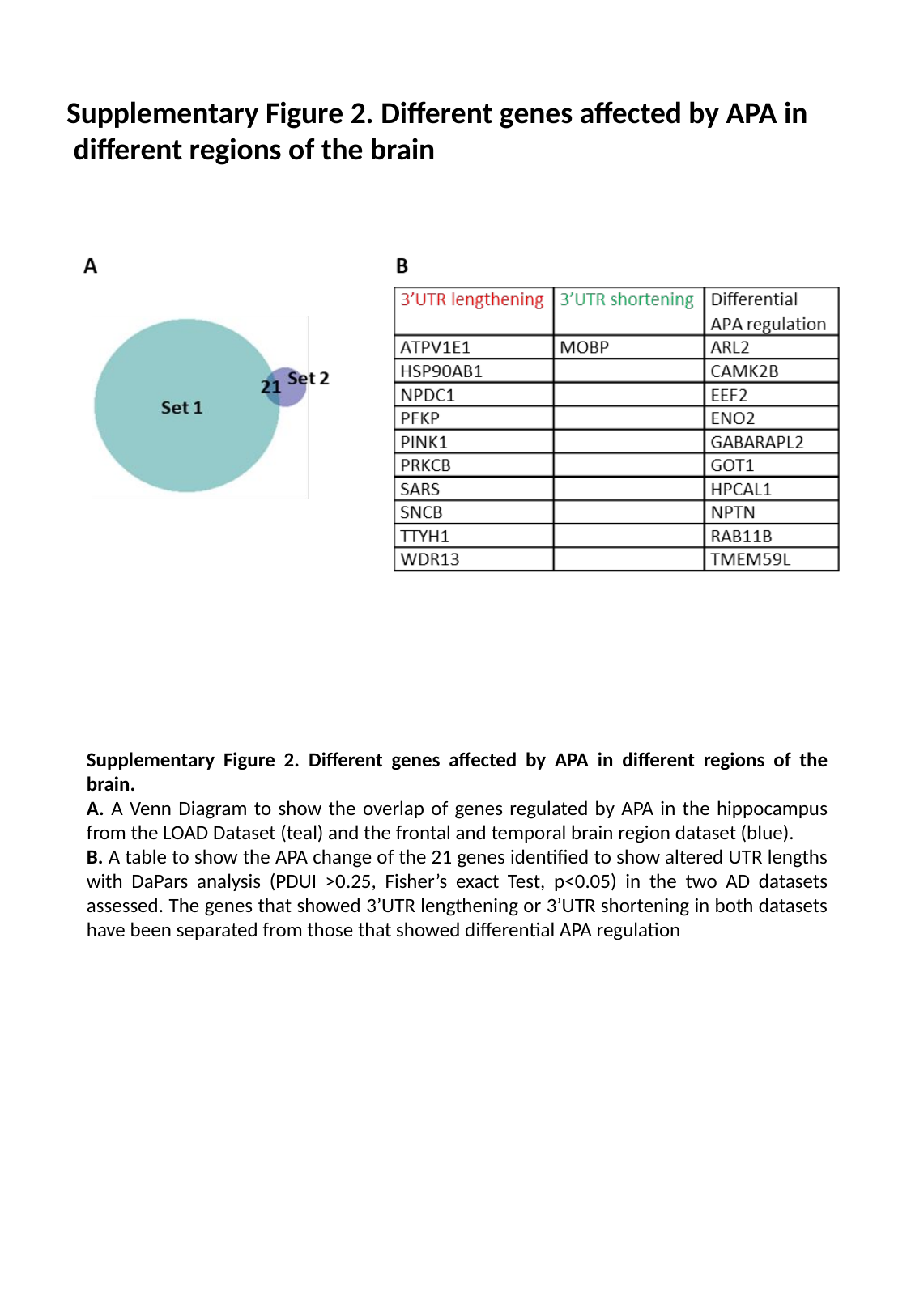

Supplementary Figure 2. Different genes affected by APA in
 different regions of the brain
Supplementary Figure 2. Different genes affected by APA in different regions of the brain.
A. A Venn Diagram to show the overlap of genes regulated by APA in the hippocampus from the LOAD Dataset (teal) and the frontal and temporal brain region dataset (blue).
B. A table to show the APA change of the 21 genes identified to show altered UTR lengths with DaPars analysis (PDUI >0.25, Fisher’s exact Test, p<0.05) in the two AD datasets assessed. The genes that showed 3’UTR lengthening or 3’UTR shortening in both datasets have been separated from those that showed differential APA regulation

## Slide 3
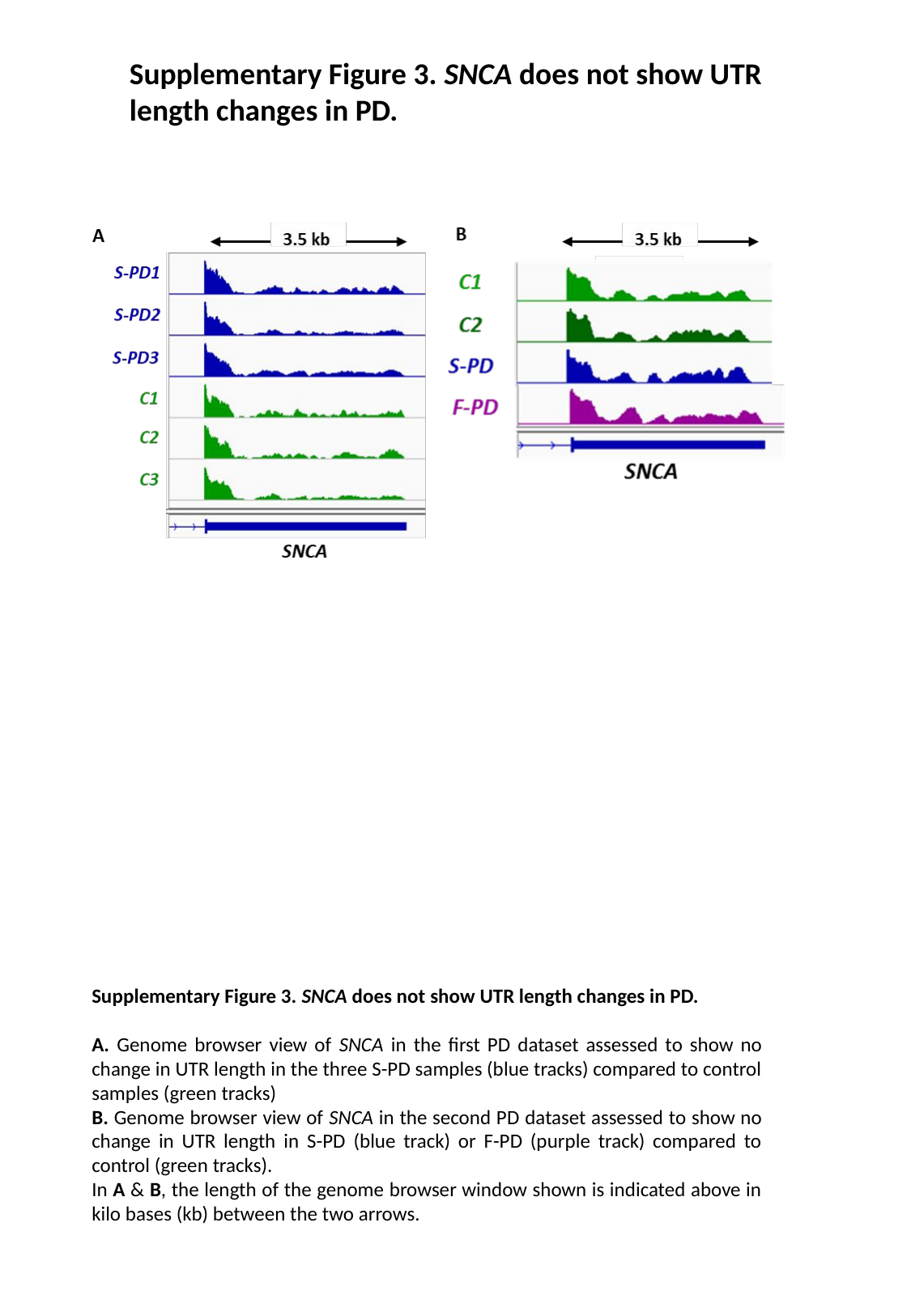

Supplementary Figure 3. SNCA does not show UTR length changes in PD.
Supplementary Figure 3. SNCA does not show UTR length changes in PD.
A. Genome browser view of SNCA in the first PD dataset assessed to show no change in UTR length in the three S-PD samples (blue tracks) compared to control samples (green tracks)
B. Genome browser view of SNCA in the second PD dataset assessed to show no change in UTR length in S-PD (blue track) or F-PD (purple track) compared to control (green tracks).
In A & B, the length of the genome browser window shown is indicated above in kilo bases (kb) between the two arrows.

## Slide 4
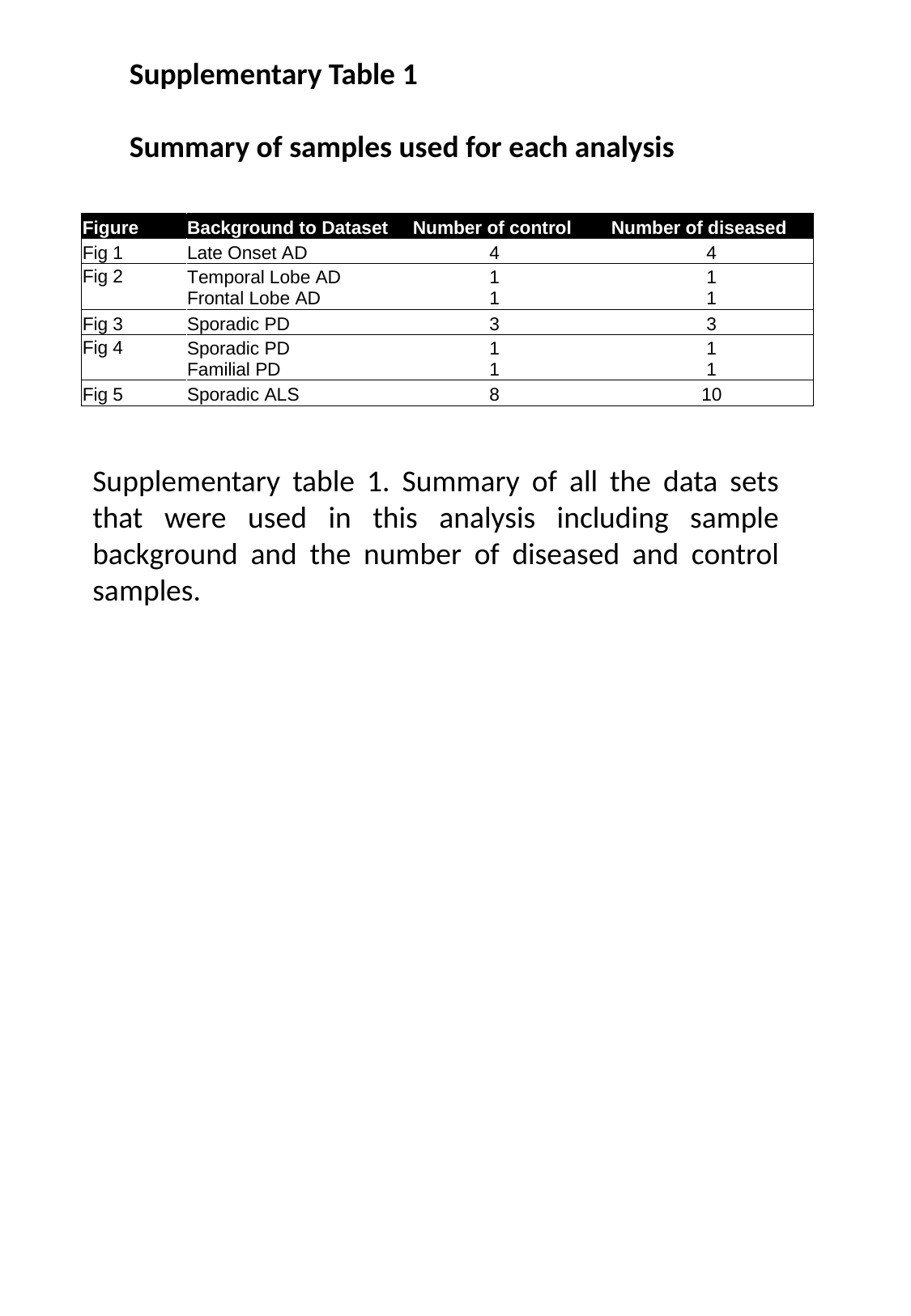

Supplementary Table 1
Summary of samples used for each analysis
Supplementary table 1. Summary of all the data sets that were used in this analysis including sample background and the number of diseased and control samples.
